# Supplementary material for: Implementation of a cylindrical distribution function for the analysis of anisotropic molecular dynamics simulations
Source: PLoS One. 2022 Dec 30;17(12):e0279679. doi: 10.1371/journal.pone.0279679 (PMC9803122; doi:10.1371/journal.pone.0279679)
Supplement: S1 File — (DOCX) [file pone.0279679.s001.docx]

**S1 File**

1. **Methods**

Fully atomistic molecular dynamics (MD) simulations were performed in Gromacs 2019.2 [8, 24-29] on the ARC3 machine at the University of Leeds, with support for NVIDIA P100 GPUs through CUDA 10.1.168. For all molecules we used the General Amber Force Field (GAFF), [50] with modifications for liquid crystalline molecules (GAFF-LCFF), as this gives significant improvements over conventional GAFF in terms of predicting heats of formation and density, but also in term of its ability to generate thermodynamically stable liquid crystalline order.

Topologies were generated using AmberTools 16 [51, 52] and converted into Gromacs readable format with Acpype. [53] Atomic charges were determined using the RESP method [54] for geometries optimised at the B3LYP/6-31G(d) level of DFT [55, 56] using the Gaussian G09 revision d01 software package. [57]

For simulations of liquid crystalline materials (with the exception of HAT6) we constructed initial low density lattices with random positional and orientational order. For 5CB we simulated 3600 molecules, for RM554 we simulated 680 molecules, for C5-Ph-ODBP-Ph-OC12 we simulated 600 molecules, for 8OCB we simulated 1500 molecules. Following energy minimisation, the simulation box was then rapidly compressed with an isotropic pressure of 100 bar for a total of 5 ns, yielding a mass density of ~ 1 g cm^3^ which is typical of that of low molecular weight liquid crystals. Following compression, simulations were equilibrated for 10 ns with an isotropic barostat (1 bar) and a temperature of 500 K to yield an isotropic starting configuration.

For RM554 a short biasing simulation was used to enforce polar order; a static electric field (0.5 V nm^-1^) was applied along one of the Cartesian axes of the simulation for 10 ns, generating a polar nematic starting configuration. The biasing simulation was performed at a temperature of 375 K with an isotropic barostat (P = 1 Bar).

For HAT6 we observed that starting from an isotropic configuration yielded randomly oriented columns of molecules that entangle, and fail to converge to give a regular columnar lattice. We therefore started from a *pseudo* columnar starting configuration, whereby 502 molecules of were placed on a hexagonal grid with lateral (x, y) separation of 3 nm and vertical (z) separation of 0.6 nm in a 11x11x14 nm box. Following energy minimization we perform a short compression simulation with an anisotropic pressure of 100 Bar before performing the production MD run at a temperature of 330 K for 100 ns.

Production MD runs of a further 250 ns (50 ns for 5CB) were then conducted with fully anisotropic pressure coupling (1 Bar) at the indicated temperatures; analysis was performed on production MD run only. In the case of 8OCB the simulation was performed for 430 ns, and only frames corresponding to the SmA phase were used in CDF analysis (~180 ns onwards).

A time step of 0.5 fs was used, and trajectories were recorded every 50 ps. Simulations employed periodic boundary conditions in xyz. Bonds lengths were constrained to their equilibrium values with the LINCS algorithm [58]. During production MD simulations the system pressure was maintained at 1 Bar using an anisotropic Parrinello-Rahamn barostat, [59, 60] the relative box dimensions to independently vary in all dimensions. Compressabilities in xyz dimensions were set to 4.5e-5, with the off-diagonal compressibilities were set to zero to ensure the simulation box remained rectangular. Simulation temperature was controlled with a Nosé–Hoover thermostat. [61, 62] Long-range electrostatic interactions were calculated using the Particle Mesh Ewald method with a cut-off value of 1.2 nm. A van der Waals cut-off of 1.2 nm was used. MD trajectories were visualised using PyMOL 4.5. Q-tensor analysis was performed using MDTraj 1.9.8. [23]

**References**

50. J. Wang, R. M. Wolf, J. W. Caldwell, P. A. Kollman and D. A. Case, 2004, **25**, 1157-1174.

51. D. A. Case, T. E. Cheatham Iii, T. Darden, H. Gohlke, R. Luo, K. M. Merz Jr, et al. *J Comput Chem*, 2005, **26**, 1668-1688.

52. J. Wang, W. Wang, P. A. Kollman and D. A. Case, *Journal of Molecular Graphics and Modelling*, 2006, **25**, 247-260.

53. A. W. Sousa da Silva and W. F. Vranken, *BMC Research Notes*, 2012, **5**, 367.

54. C. I. Bayly, P. Cieplak, W. Cornell and P. A. Kollman, *The Journal of Physical Chemistry*, 1993, **97**, 10269-10280.

55. C. Lee, W. Yang and R. G. Parr, *Phys Rev B*, 1988, **37**, 785-789.

56. A. D. Becke, 1993, **98**, 5648-5652.

57. M. J. Frisch, G. W. Trucks, H. B. Schlegel, G. E. Scuseria, M. A. Robb, J. R. Cheeseman, et al. *Gaussian 09*, 2009.

58. B. Hess, H. Bekker, H. J. C. Berendsen and J. G. E. M. Fraaije, *J Comput Chem*, 1997, **18**, 1463-1472.

59. M. Parrinello and A. Rahman, *J Appl Phys*, 1981, **52**, 7182-7190.

60. S. Nosé and M. L. Klein, *Mol Phys*, 1983, **50**, 1055-1076.

61. S. Nosé, *Mol Phys*, 1984, **52**, 255-268.

62. W. G. Hoover, *Phys Rev A*, 1985, **31**, 1695-1697.
